# Supplementary material for: Risk and protective factors for drug dependence in two Moroccan high-risk male populations
Source: PeerJ. 2018 Nov 8;6:e5930. doi: 10.7717/peerj.5930 (PMC6230435; doi:10.7717/peerj.5930)
Supplement: Supplemental Information 2 [file peerj-06-5930-s002.pdf]

# خلفية المشاركين

| | |
|---|---|
| العمر أو تاريخ الازدياد | |

- ما هو وضعك العائلي: (ضع علامة X أمام الإجابة المناسبة)

☐ أعزب ☐ منفصل ☐ شريك ☐ مطلق ☐ متزوج ☐ متزوج للمرة الثانية

| | | |
|---|---|---|
| هل عندك أطفال | نعم | لا |

# تعليم

| | |
|---|---|
| متى تركت المدرسة؟ ( السن و المستوى الدراسي) | |

ما هو أعلى مستوى من التعليم حققته ؟

☐ دون الحصول على مؤهلات ☐ مستوى ابتدائي ☐ مستوى إعدادي ☐ مستوى ثانوي ☐ التعليم العالي

| | | |
|---|---|---|
| هل لديك مهنة أو تجارة ؟ | نعم | لا |
| هل كنت تتوفر على عمل خلال العام الذي سبق دخولك لسجن؟ | نعم | لا |

# السجل النفسي

- هل سبق أن تم تشخيصك بأي من الأمراض الآتية , من طرف اخصائى

| | | | |
|---|---|---|---|
| كآبة | نعم | لا | لا أعرف |
| اضطرابات القلق | نعم | لا | لا أعرف |
| اضطراب الوسواس القهري | نعم | لا | لا أعرف |
| اضطراب ما بعد الصدمة | نعم | لا | لا أعرف |
| اضطراب ثنائي القطب | نعم | لا | لا أعرف |
| اضطرابات الأكل | نعم | لا | لا أعرف |
| انفصام الشخصية | نعم | لا | لا أعرف |
| اضطراب الشخصية | نعم | لا | لا أعرف |
| اضطرابات استخدام المواد (الكحول أو المخدرات) | نعم | لا | لا أعرف |
